# A Conservative Approach to Inflammatory Myofibroblastic Tumor of the Bladder: A Case Report and Review of Literature

**DOI:** 10.1155/2021/6660356

**Published:** 2021-03-08

**Authors:** Rawad Abou Zahr, Elie Ghabi, Mehdi Idrissi-Kaitouni, Thierry Roumeguere

**Affiliations:** ^1^Urology Department, Erasme Hospital, Université Libre de Bruxelles, Brussels, Belgium; ^2^Urology Department, Saint George Hospital University Medical Center, University of Balamand, Beirut, Lebanon

## Abstract

Inflammatory myofibroblastic tumors (IMTs) are particularly rare tumors that have been described in various anatomic locations, of which the urinary bladder is the most common. These benign tumors are amendable to conservative therapy but are notoriously difficult to diagnose given their mimicry of malignant sarcomas and sarcomatoid carcinomas, making an accurate diagnosis paramount to spare a patient radical and unnecessary treatment. We hereby present the case of a 37-year-old female patient who was diagnosed with an IMT of the urinary bladder during workup for painless gross hematuria. Patient was successfully managed with a laparoscopic partial cystectomy and is free of recurrence 5 years after surgery. IMTs are rare benign tumors that share the same clinical presentation as malignant bladder tumors. Deep biopsy and experienced pathologist are crucial in establishing diagnosis and avoiding patient radical treatment. This case is a classical demonstration of a remarkably rare tumor that was adequately managed with conservative therapy, achieving excellent clinical outcomes.

## 1. Introduction

Inflammatory myofibroblastic tumors (IMTs) are particularly rare tumors that have been described in various anatomic locations such as the lungs, pancreas, bladder, and prostate [1–5]. First described by Roth in 1980, this rare entity came to be commonly reported in urinary bladders and the lungs as the most common sites [1, 4]. These tumors are characterized by a spindle cell proliferation loosely distributed in a congested myxoid stroma with variable populations of inflammatory cells [1, 3, 4]. IMTs share similar features with sarcomas and sarcomatoid carcinomas which makes the diagnosis of IMTs challenging, as these tumors tend to present with similar clinical presentation as urothelial bladder tumors. Hence, an accurate diagnosis is critical as IMTs have a benign course, are unlikely to recur or metastasize, and are amendable to conservative therapy [1, 3]. Our case is that of a 37-year-old female patient who was diagnosed with an IMT during workup for painless gross hematuria. She was successfully managed with a laparoscopic partial cystectomy and is free of recurrence 5 years postoperatively.

## 2. Case Presentation

**Figure 1:**
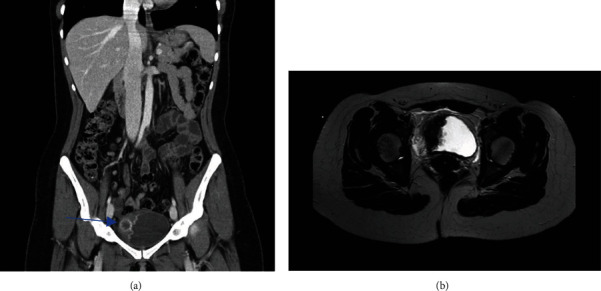
Coronal CT scan (a) and axial MRI (b) demonstrating 2 × 2 cm exophytic polyp (arrow) associated with significant bladder wall thickening of the urinary bladder with invasion of detrusor muscle.

A 37-year-old female, nonsmoker, presented to our institution for painless gross hematuria. She had no previous history of abdominopelvic surgery, urinary or gynecologic instrumentation, nor any history of recurrent or chronic urinary infections. Physical examination and serum biochemical investigations were unremarkable. Urine analysis and microscopy revealed numerous morphologically normal red blood cells with the presence of inflammatory cells. Significant wall thickening of the right lateral wall of the urinary bladder centered around a 2 × 2 cm exophytic mass was seen on computed tomography (CT) scan, as seen in Figure 1. Further assessment was done using magnetic resonance imaging (MRI) that revealed tumoral extension into the detrusor muscle. No pelvic or retroperitoneal lymphadenopathy was noted. A transurethral deep biopsy of the tumor was performed.

**Figure 2:**
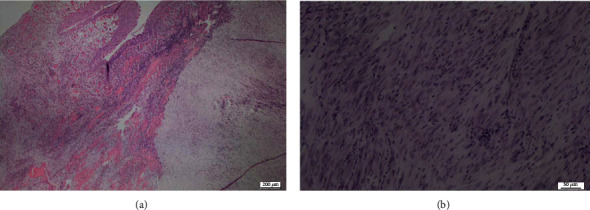
Wright and Giemsa stain demonstrating fusiform spindle cells with cellular atypia and an edematous myxoid stroma under low (a) and high power (b) magnification.

Histologic analysis of the resection fragments revealed evidence of ulcerations in the urothelium with submucosal proliferation of fusiform spindle cells with mild cytonuclear atypia and minimal mitotic activity in a richly vascularized and edematous myxoid stroma. Certain foci of necrosis were noted. A dense inflammatory infiltrate composed of lymphocytes, polynuclear neutrophils, and polynuclear eosinophils was present in the tumoral tissue (Figure 2). Immunohistochemistry was positive for AE1/AE3 along the entire urothelium and less so in the spindle cell foci and positive for SMA in the spindle cell foci.

**Figure 3:**
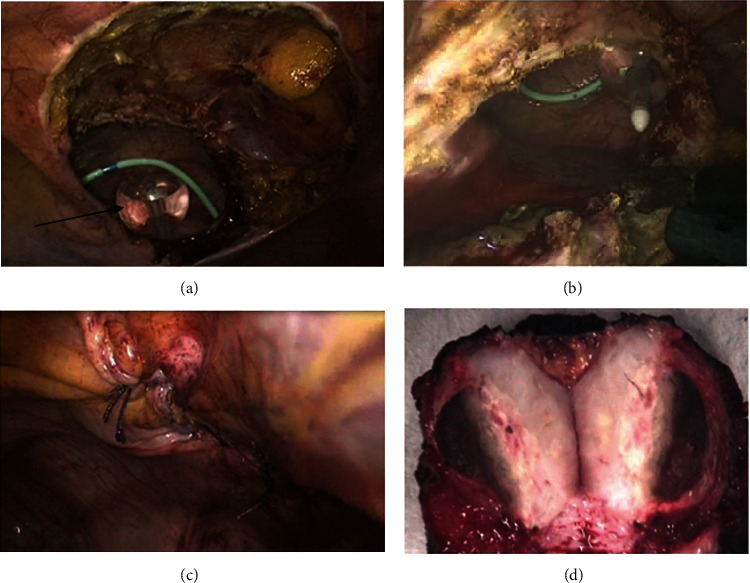
Intraoperative view of the bladder defect post partial cystectomy, arrow on foley catheter (a and b), view of the bladder post defect repair (c), and a cross-section of the tumor revealing a white soft myxoid mass (d).

Given the histologic features and the immunohistochemical profile, diagnosis of a pseudosarcomatous myofibroblastic tumor of the urinary bladder was made. The patient successfully underwent a laparoscopic partial cystectomy to resect the remaining tumor mass (Figure 3). Histologic evaluation revealed similar findings with negative resection margins. No adjuvant treatment was offered. Close follow-up by yearly CT scan imaging and biannual cystoscopy revealed no evidence of disease recurrence 5 years postoperatively.

## 3. Discussion

Nonepithelial tumors of the urinary bladder make up 2% to 5% of primary neoplasms of the bladder [6]. Inflammatory myofibroblastic tumors belong to this class of neoplasms, and though numerous cases have been described yet the incidence of these rare tumors is largely unknown. They are observed across all ages but generally occur in children and young adults, with most reported cases belonging to the pediatric population, and with a slight female predominance [1, 3, 6]. IMTs are associated with smoking, prior abdominopelvic surgery, and bladder instrumentation, yet cases with no discernable risk factors have also been described [6, 7].

Pathologically, IMTs are described as gray-white or tan-yellow firm well-circumscribed lesions with sizes ranging from 1 to 20 cm and a variable degree of focal necrosis, hemorrhage or cystic changes, or calcification [8]. A cross-section of the tumor seen in our case is similar to the classical appearance recognized in the literature. Histologically, the tumor is characterized by a dense proliferation of myofibroblast and fibroblast spindle cells in an edematous myxoid stroma with a large infiltrate of inflammatory cells predominantly composed of lymphocytes, neutrophils, and eosinophils [8]. The histologic features may however vary between tumors at the same site [4]. Three histologic patterns are generally recognized, the first has the appearance of granulation tissue and nodular fasciitis characterized by spindle cells in a myxoid pattern with an abundant eosinophilic cytoplasm [4, 8]. The second pattern involves densely packed spindle cells with a more prominent inflammatory component and the third is relatively hypocellular with an abundance of collagen, plasma cells, lymphocytes, and eosinophils [4, 8]. These patterns, however, are similar to those seen in malignant sarcomas and spindled carcinomas such as leiomyosarcoma, sarcomatoid carcinoma, and rhabdomyosarcoma [8]. Importantly, low degrees of cellular and nuclear atypia and a low number of mitotic figures are important distinguishing factors that may help in diagnosing an IMT [6].

The immunohistochemical profile of IMTs often overlaps with other malignancies, which makes the diagnosis of an IMT more difficult. In general, IMTs stain positive for vimentin, desmin, smooth muscle actin, muscle-specific actin, and keratin [1]. ALK positivity is recognized as a particularly useful and specific immunological marker for IMTs [1]. IMTs also characteristically stain negative for myogenin (Myf4) and MyoD1 which are generally positive in embryonal rhamdomyosarcomas, a malignant soft tissue tumor that shares similar histologic features with IMTs [1].

Clinically, IMTs are slow-growing benign tumors that are amendable to conservative treatment [1, 3]. Generally, management is surgical with complete resection with negative margins being critical. Hence, conservative management can consist of a partial cystectomy preserving the quality of life of the patient and avoiding the complications and morbidity of radical cystectomy. A limited role for chemotherapy exists; they are almost exclusively used in the management of unresectable tumors, incomplete resections, or if resections would lead to significant morbidity [1, 2]. IMTs generally do not recur following complete resection; however, recurrence in genitourinary cases has been observed in up to 2.7% of patients [1]. Timely follow-up is therefore crucial with imaging or endoscopy as appropriate, to identify recurrent disease or possible metastasis.

## 4. Conclusion

In conclusion, inflammatory myofibroblastic tumors are remarkably rare nonepithelial benign neoplasms of unknown pathogenesis that presents similarly to malignant diseases. These tumors present a diagnostic challenge due to the overlap of histologic and immunohistochemical features seen in malignant neoplasms such as leiomyosarcoma, embryonal rhabdomyosarcoma, carcinoid sarcoma, and other malignant soft tissue neoplasms. Certain histologic and immunohistochemical findings are somewhat specific to IMTs which aids in differentiating them. A deep transurethral resection biopsy and an experienced pathologist are paramount in establishing the diagnosis and avoiding the patient radical treatments. The standard of care is surgical resection with negative margins with chemotherapy and radiotherapy reserved for unresectable or incompletely resected tumors. These tumors generally do not recur or metastasize; however, cases of recurrence or metastasis have been reported. Our case is a classical demonstration of an IMT discovered in a 37-year-old female managed with a laparoscopic partial cystectomy and remains recurrence free after 5 years.

## Data Availability

The data used to support the findings of this study are included within the article.
